# Evidence-based general practice: Selected abstracts from the 98th EGPRN Meeting, Porto, Portugal, 9–12 May 2024: **Evidence-based general practice**

**DOI:** 10.1080/13814788.2024.2390444

**Published:** 2024-08-27

**Authors:** Mine Kaya Bezgin

## Abstract

With its strong commitment to Primary Care, Portugal’s healthcare landscape has witnessed significant strides in recent years. The 98th EGPRN meeting in Porto was a testament to the dedication and progress of primary care research and education in the country.The theme of the 98th EGPRN meeting, ‘Evidence-Based General Practice’, aligned seamlessly with the evolving healthcare landscape in Portugal. This paradigm shift aims to foster better interdisciplinary collaboration and establish a framework for connecting welfare and healthcare on a local community level. Over the past few decades, primary health care and general practitioners in Portugal have gained prominence, following the guidelines set forth by the World Health Organisation (WHO). With a focus on prevention and wellbeing, practices have transitioned into multidisciplinary health centres. Considering this evolving healthcare context, the need for innovative and multidisciplinary research designs becomes imperative for translating evidence into practice. Moreover, this gathering provided an opportunity to discuss the challenges and opportunities associated with population-based studies. Understanding the collective health needs of communities and implementing data-driven strategies will shape the future of evidence-based general practice in Europe.In conclusion, the 98th EGPRN meeting in Porto, Portugal, was a dynamic platform for exchange, learning, and innovation, as we collectively strive to enhance evidence-based general practice. As an academic community, we aim to chart a course towards a healthier and more interconnected future for primary care, leaving a positive impact on the wellbeing of communities and patients alike.

