# Supplementary material for: Evidence-based general practice: Selected abstracts from the 98th EGPRN Meeting, Porto, Portugal, 9–12 May 2024: Evidence-based general practice
Source: Eur J Gen Pract. 2024 Aug 27;30(1):2390444. doi: 10.1080/13814788.2024.2390444 (PMC11351361; doi:10.1080/13814788.2024.2390444)
Supplement: Related texts

# Abstract: Who wants to work 27 hours a day? – why research and guidelines should consider the clinician time needed to treat (TNT)

**Authors:** Minna Johansson

**Affiliations:** Herrestads Healthcare Centre, Närhälsan Director, Global Center for Sustainable Healthcare Researcher, Gothenburg University, FoUUI Fyrbodal, Cochrane, Sweden

## Abstract

This lecture argued that the way we currently practice medicine is unsustainable for patients, clinicians, health systems, our societies and the planet. We need a new way to appreciate human, financial and environmental resources in healthcare – we need to understand what we do in medicine through a lens of sustainability.

For example, there is a massive mismatch between what is being recommended in clinical practice guidelines and the available time for clinicians to implement the recommended care. To follow current guidelines, US primary care physicians are estimated to require 27 hours per working day. Just to implement the European hypertension guidelines, Norway would need more general practitioners than currently in practice. More physicians (from all specialties) and five times as many nurses as currently available may be needed to implement all lifestyle interventions recommended by the National Institute of Health and Care Excellence in the United Kingdom. Were clinicians to follow these guidelines, there would be no time left to care for other ill patients and the healthcare system would collapse.

It is thus evident that clinicians face a barrage of recommendations that in total are impossible to implement. As a result, clinicians must prioritise which recommendations to follow in which patients. Without guidance, and under the pressure of time, prioritising decisions at the point of care will be implicit, variable, and likely often misguided.

One – at least partial – solution to this problem would be for guideline panels to consider the time needed to implement a recommendation when determining the direction and strength of recommendations. This requires a new methodology that highlights time constraints and provides a structure for their consideration. The Time Needed to Treat (TNT) method was introduced in The BMJ in 2023. TNT provides such a structure for how guideline panels can consider the time needed to implement the recommended care. The ultimate goal of estimating TNT is to avoid clinicians and patients spending their limited time together on recommendations with smaller rather than greater importance to the individual patient, as well as to improve access to care for patients with the greatest need of medical attention.

# Abstract: Evidence-based general practice and patient-centredness

**Authors:** Bruno Heleno

**Affiliations:** Nova Medical School Lisbon and General Practitioner at USF das Conchas in Lisbon

## Abstract

This lecture covered patient stories to highlight the dual challenges and opportunities in evidence-based general practice. Like many clinicians, we navigate the tension between adhering to patient-centred care and evidence-based principles within a pay-for-performance framework. However, by returning to the original foundations of evidence-based practice—combining high-quality evidence, clinical expertise, and patient preferences—we find a way to bridge this gap. Key components of the patient-centred clinical method, such as ‘exploring health, disease, and illness’ and ‘understanding the whole person,’ are instrumental in applying evidence-based practice effectively. Similarly, the 5A's of evidence-based practice—assess, ask, acquire, appraise, and apply—enrich our discussions and aid in ‘finding common ground’ with our patients.

The challenge intensifies with the increasing interference of vested interests in clinical autonomy. Commercial influences, for instance, significantly sway the production of evidence, affecting which diseases are prioritised and how interventions are evaluated. Moreover, the rise of structured templates, point-of-care prompts, and pay-for-performance incentives often shifts the focus from patient care to management targets. Nonetheless, evidence-based practice remains a powerful tool against these pressures. It equips us with the critical thinking skills needed to question the relevance and robustness of evidence and to ensure our clinical decisions align with patient needs.

In summary, evidence-based practice serves as a foundation for not only addressing the challenges we face but also advancing healthcare in a way that truly benefits patients. It promotes a critical evaluation of evidence and a commitment to patient-centred care, offering tools to resist commercial and unreasonable managerial pressures. Notable advances in the last 30 years are a broader sense of what constitutes evidence, how to ensure that the voices of patients shape which evidence is produced, and more transparent methods of translating research findings into clinical recommendations. We still need evidence about better ways of understanding each patient as a whole person, or of finding common ground. These are great opportunities for general practice to be even more evidence based.

# Abstract: Decision aids in primary healthcare – needs and experiences

**Authors:** Mafalda Proença-Portugal, Bruno Heleno, Sónia Dias, Ana Gama, Sofia Baptista

**Affiliations:** a USF da Baixa, ULS São José, Lisboa, Portugal; b NOVA Medical School, Universidade NOVA de Lisboa, Lisboa, Portugal

**Keywords:** Decision aids, focus groups, primary healthcare, shared decision making, qualitative study

## Abstract

**Background:**

Decision aids (DA) are evidence-based tools that support shared decision-making, promoting an active role of patients in health decisions. Portuguese clinical guidelines are tools for clinical decision-making but have low incorporation of patient’s values and perspectives. However, the complexity of available information and the ambiguity between choices call for the patients’ participation in the decision-making process. DA adapted to the Portuguese reality could be useful in primary healthcare (PHC).

**Research questions:**

To explore the perception of health professionals about DA and their utility within the scope of PHC in Portugal; to identify barriers and facilitators to the implementation of DA in clinical practice; and to identify clinical topics that would benefit from translation and cultural adaptation.

**Method:**

Qualitative study. Seven focus groups were held with 33 general practitioners and residents (more than 6 months of residency). Discussions were recorded, transcribed, and anonymised, then thematically analysed by two authors independently. The Regional Health Administration of Lisbon and Tagus Valley Health Ethics Committee approved the study.

**Results:**

Most participants were not familiar with the concept of DA. All participants mentioned that DAs support the professional’s clinical decision and only one mentioned their potential to support shared decision-making with their patients. Participants generally revealed a positive attitude towards the use of DA. Concerns surrounded the lack of funding and the need for longer consultation time. Participants mentioned as facilitators: the possibility of integrating these tools into computer systems and their translation and adaptation to the Portuguese population. Younger and more literate patients were the most receptive to DA. Clinical topics of interest included screening, diagnosis and treatment.

**Conclusions:**

Despite being unfamiliar to most participants, integrating DA in PHC was well received and these may provide a potential added value regarding the provision of healthcare to patients

# Abstract: Factors-influencing primary care physicians recommending patients to use digital health technologies for self-management: A cross-sectional study across 20 countries

**Authors:** Ana Luisa Neves, Insight Collaborative Research Team

**Affiliations:** Global Digital Health Unit, Imperial College London, Porto, Portugal

## Abstract

**Background:**

Expanding access to self-management via Digital Health Technologies may supplement traditional care, mitigating pressures on primary care through self-management. Primary Care Physicians can play a critical role in the integration of digital health technologies into patient care, but it is unclear what factors influence Primary Care Physicians’ recommendation of such technologies.

**Research questions:**

To identify the factors associated with Primary Care Physicians recommending digital health technologies to patients for self-management before and during the pandemic.

**Method:**

Primary Care Physicians across 20 countries completed an online questionnaire between June and September 2020. The outcome was a self-report recommending patient to at least one of six listed forms of digital health technologies (symptom checker/self-assessment tools, online information resources, health trackers, mindfulness apps, online counselling and crisis resolution services). Multivariate logistic regression models were performed to identify factors associated with recommending digital health technologies to patients.

**Results:**

A total of 1,592 Primary Care Physicians were included. Before the pandemic, the odds of recommending digital health technologies for self-management were lower for Primary Care Physicians not involved in teaching (aOR 0.64, 95%CI 0.51–0.8), or practising in Turkey, Australia, Chile, Colombia, France, Italy, Poland, Portugal, Slovenia, and Spain (aORs range: aOR 0.18, 95%CI 0.1–0.34 [Turkey]; aOR 0.58, 95% CI 0.36–0.93) [Australia]). There was no significant difference in recommending digital health technologies before and during the pandemic (53.2% vs 54.7%, *p* = 0.215).

**Conclusions:**

Involvement in teaching (pre-pandemic) and practising in a rural setting (during the pandemic) positively influenced the recommendation of digital health technologies. Significant variation in recommending digital health technologies was present across countries. Further research is indicated to better understand potential drivers of variation, including characteristics of the populations served, as well as national health systems and policies.

# Abstract: Improving medication review and cardiovascular risk management in patients using antipsychotics in general practice – a pilot study

**Authors:** Karlijn Van Den Brule-Barnhoorn, Kirsti Jakobs, Sietske Grol, Jan Van Lieshout, Marion Biermans, Erik Bischoff

**Affiliations:** Radboudumc, Nijmegen, Netherlands

**Keywords:** Atypicial antipsychotic medication, cardiovascular risk, medication review, transmural, multidisciplinary

## Abstract

**Background:**

Patients using atypical antipsychotic medication (APM) have a significantly increased cardiovascular risk (CVR). However, monitoring of CVR in general practice (GP) is insufficient due to a lack of knowledge and time. TACTIC is a one-time transmural intervention aimed at reducing inappropriate APM use and CVR in patients in GP. TACTIC consists of an information meeting, a multidisciplinary meeting with the patient, and a follow-up consultation with the GP in which an individualised treatment plan is drawn up. We investigated the feasibility of TACTIC in a qualitative pilot study to optimise the intervention for application in an upcoming RCT.

**Research questions:**

What are the barriers and facilitators for the feasibility of TACTIC according to patients and healthcare professionals? What are the suggestions for improvement for the intervention?

**Method:**

Patients, selected through purposive sampling, were invited for individual interviews. Healthcare professionals were invited for focus group interviews. Interviews were semi-structured and interview guides were based on the Normalisation Process Theory. We used the Framework Method for analysis of our data, to identify themes. We applied the Consolidated criteria for Reporting Qualitative research (COREQ) guidelines.

**Results:**

We conducted eight individual and two focus group interviews, with 11 healthcare professionals in total. This resulted in several important facilitating factors: a personal approach, a clear information meeting, and a summary with advice from the psychiatrist after the multidisciplinary meeting. Barriers appeared to be the high workload for GPs when recruiting participants, the relatively short duration of the multidisciplinary meeting and the tension/anxiety patients felt during participation. Suggestions for improvement included indication, management of patients’ expectations, communication and data flow between healthcare professionals.

**Conclusions:**

This qualitative analysis uncovered various barriers and facilitators, and useful suggestions for improvement, which we will use to further, develop the TACTIC intervention in preparation for the upcoming RCT.

# Abstract: General practitioners’ approach to sexuality over 60: A mixed study

**Authors:** Maxime Pautrat

**Affiliations:** Maison de Santé Pluridisciplinaire, Médecine Générale, Ligueil, France

**Keywords:** Sexuality, elderly, health communication, general practitioners

## Abstract

**Background:**

The sexuality of the elderly is the focus of recent research, but still rarely discussed during primary care consultations.

**Research questions:**

This study aimed to describe the practice of general practitioners (GPs) and to explore what GPs think about communicating about the sexuality of elderly people (EP).

**Method:**

Mixed study included quantitative and qualitative study, carried out between June 2021 and June 2022 among GPs. The quantitative study included a descriptive analysis, based on an anonymous questionnaire designed by the investigators and distributed by mailing via professional structures. It included the usual demographic data completed with questions about sexuality. The qualitative study was an inductive analysis inspired by grounded theory. It aimed to conceptualise the representations of GPs recruited by a snowball effect, based on individual semi-directed interviews.

**Results:**

One hundred and five questionnaires were analysed. There was a significant link between male gender and comfort (*p* < 0.05). GPs who were ‘at ease’ were significantly older than those who were ‘not at ease’ (*p* < 0.05). Twelve GPs were interviewed. During the interviews, they reaffirmed their role as being ‘in the best place’ while expressing their ‘discomfort’. A patient-centred approach was favoured, with a ‘trusting’ approach that went beyond ‘Judeo-Christian modesty’. The GPs reported a transference phenomenon linked to their personal and professional experience. ‘Resistance, it’s simple, it’s always on the doctor’s side’ testified to their reflexivity.

**Conclusions:**

GPs’ perceptions of how to deal with the sexuality of EP led to the emergence of new proposals, such as not limiting sexuality to coitus, combating societal taboos, and promoting mutual support in peer groups. This study opens other avenues for reflection, such as screening for even old cases of violence and sexually transmitted infections, which are often overlooked in the over-60s.

# Abstract: Eurodata study: The role of primary health care during COVID-19 in Europe

**Authors:** Sara Ares Blanco, Marina Guisado Clavero, Ileana Gefaell, Lourdes Ramos Del Rio, Kathryn Hoffmann, Elena Brutskaya-Stempkovskaya, Marijana Jandrić-Kočić, Radost Assenova, Goranka Petricek, Miroslav Hanževački, Asja Ćosić Divjak, Heidrun Lingner, Theresa Sentker, Philippe-Richard J. Domeyer, Maria Bakola, Peter Torzsa, Ábel Perjés, Louise Fitzgerald, Limor Adler, Shlomo Vinker, Ferdinando Petrazzuoli, Alice Serafini, Murauskienė Liubovė, Martin Sattler, Maryher Delphin Peña, Aleksandar Kirkovski, Dragan Gjorgjievski, Katarzyna Nessler, Anna Krzton-Krolewiecka, Carmen Busneag, Snežana Knežević, Milena Kostić, Davorina Petek, Büsra Çimen Korkmaz, Erva Kirkoç, Оксана Ільков, Shushman Ivanna, Bruno Heleno, Ana Luísa Neves, Bert Vaes, Sherihane Bensemmane, Anna Segernäs, Naldy Parodi, Sven Streit, Senn Oliver, Raquel Gómez Bravo, María Pilar Astier Peña

**Affiliations:** Eurodata Collaborative Group Spanish Society of Family and Community Medicine semFYC, Madrid, Spain

**Keywords:** COVID-19, primary health care, Europe, population surveillance, clinical pathways, health status indicators, apps, vaccination

## Abstract

**Background:**

Most COVID-19 cases received care exclusively through Primary Health Care (PHC). Although the role of PHC during the pandemic has been described in certain aspects, it has not been comprehensively covered for all areas where PHC operated in Europe.

**Research questions:**

What was the role and visibility of PHC during the pandemic in Europe?

**Method:**

Descriptive, cross-sectional, retrospective study involving 27-31 European countries. Qualitative data were gathered through a semi-structured questionnaire to assess PHC's role in COVID-19 pathways, Long Term Care Facility (LTCF), indicators, apps, vaccination, and immunisation information systems. A Delphi study was conducted to define COVID-19 indicators for PHC. Data collection spanned March 2020-December 2021.

**Results:**

In 27 countries, PHC served as the entry point for COVID-19 patients, while Telehealth services were conducted in 30 countries. There was no consensus on the isolation duration, and General Practitioners (GPs) issued sick leaves in 21 countries. Diagnostic resources and treatments varied across regions. PHC provided COVID-19 care in 22 countries, with LTCF nurses coordinating care in 19 countries. Only 13 countries collected PHC activity indicators, and 5 shared the same indicator (total COVID-19 cases in PHC). A Delphi study identified the preferred indicator as COVID-19 cases in PHC/total respiratory infection cases. Five countries integrated the COVID-19 app with PHC information systems. COVID-19 vaccines were administered in PHC in 25 countries, with nurses and GPs administering them in 25 and 21 countries, respectively. PHC actively participated in vaccine communication campaigns in 10 countries, showing heterogeneity in priority group criteria and vaccine types. Finally, 19 countries integrated Immunisation Information Systems into PHC.

**Conclusions:**

PHC had a relevant role in COVID-19 pandemic but heterogeneity has been described in PHC managing COVID-19 pandemic across European countries. Specific indicators from PHC might be included in future pandemics.

# Abstract: Frailty and its association with long-term mortality among community-dwelling older adults aged 75 years and over

**Authors:** Maor Lewis, Galia Zacay, Anthony Heymann, Dan Justo

**Affiliations:** Meuhedet Health Services, Tel Aviv, Israel

**Keywords:** Community-dwelling, cumulative deficit, frailty, mortality, older adults

## Abstract

**Background:**

The prevalence of frailty in a population of community-dwelling older adults and its association with long-term mortality has not been previously studied in Israel on a large scale.

**Research questions:**

We sought to demonstrate the potential utility of a frailty index in a large database of community-dwelling older adults aged 75 years and over in Israel by studying the prevalence of frailty and its association with long-term mortality in this population.

**Method:**

A retrospective cohort study using a large Israeli Health Maintenance Organisation database. The prevalence of frailty and its association with all-cause mortality was studied among older adults aged 75 years and over who were followed for 2–8 years. The Frailty index was calculated using the cumulative deficit method including 28 chronic diseases and age-related health deficits.

**Results:**

The cohort included 43,737 community-dwelling older adults: median age was 77 years (IQR 75-82 years); 19,300 (44.1%) patients were males; most patients were non-orthodox Jews (*n* = 35,515, 81.2%). Overall, 19,396 (44.3%) older adults were frail: 12,260 (28.0%) mildly frail, 5,533 (12.7%) moderately frail, and 1,603 (3.7%) severely frail. Overall, 15,064 (34.4%) older adults died during the follow-up period: 4,782 (39.0%) mildly frail, 3,016 (54.5%) moderately frail, and 1,080 (67.4%) severely frail. Cox regression analysis showed that mortality was associated with severe frailty (HR 2.63, 95%CI 2.45-2.80), moderate frailty (HR 2.05, 95%CI 1.96-2.14), and mild frailty (HR 1.45, 95%CI 1.39-1.51), controlled for age, gender, and population sector. In patients aged 90 years and over, there were no longer differences in cumulative survival between patients with moderate and severe frailty (*p* = 0.408).

**Conclusions:**

Frailty is common among the population of community-dwelling Israeli older adults aged 75 years and over and associated with long-term mortality across frailty levels. Among older patients aged 90 years and over there are no longer differences in cumulative survival between patients with moderate and severe frailty.

# Abstract: Ukrainian refugees healthcare resource utilisation in Israel

**Authors:** Limor Adler, Eugene Merzon, Bar Cohen, Pavlo Kolesnyk, Shlomo Vinker

**Affiliations:** Family Medicine, Faculty of Medicine, Tel Aviv University, Tel Aviv, Sde Yoav, Israel

**Keywords:** Ukranian refugees, healthcare resource utilisation, primary care

## Abstract

**Background:**

Since February 2022, when the Russians invaded Ukraine and started the Russia-Ukraine war, many Ukrainians left their homes in search of safety. Israel accepted more than 14,000 Ukrainian refugees.

**Research questions:**

This study aimed to evaluate healthcare resource utilisation of Ukrainian refugees and compare it to matched Israeli individuals.

**Method:**

In this retrospective cohort study, we extracted all files from the electronic medical records of Leumit Healthcare Services (LHS). LHS insures all Ukrainian refugees 60 years or older. We then matched for each Ukrainian refugee, and Israeli individuals based on age, sex and country of origin (individuals who were born in the former Soviet Union). We compared healthcare resource utilisation with Poisson regression for count variables.

**Results:**

Among the refugee group, the mean age was 71.4 (± 7.095) and the majority were females (77.37%). Compared to their matched controls, the refugees were much less likely to have mail and video visits to the doctor (IRR = 0.457 and 0.329, respectively, *p*-value = 0.00), and somewhat less likely to have a phone or regular visit (IRR = 0.838 and 0.888, respectively, *p*-value = 0.00). Across all but one specialty recorded in this study, refugees were less likely to have a consultation with a specialist (urogynecology, endocrinology, gastroenterology, general surgery, orthopaedic surgery, dermatology, ophthalmology neurology and psychiatry; IRR = 0.800, 0.485, 0.529, 0.542, 0.607, 0.352, 0.592, 0.716, 0.315, *p*-value = 0.00). The only exception is a consultation with a cardiologist (IRR = 1.209, *p*-value = 0.011). Additionally, refugees had fewer emergency room visits (IRR = 0.424, *p*-value = 0.00), fewer hospitalisations (IRR = 0.545, *p*-value = 0.00), shorter hospitalisations on average (IRR = 0.488, *p*-value = 0.00), fewer referrals for dialysis (IRR = 0.164, *p*-value = 0.00) and for surgery (IRR = 0.614, *p*-value = 0.00).

**Conclusions:**

Ukrainian refugees have fewer visits to primary care consultations, emergency department visits and hospitalisations. To better understand the reasons for these findings, another research with a different study design should conducted.

# Abstract: Interprofessional collaboration in the home care setting: Perspectives of people receiving home care, relatives, nurses, general practitioners, and therapists. Results of a qualitative analysis

**Authors:** Uta Sekanina, Britta Tetzlaff, Ana Mazur, Tilmann Huckle, Anja Kühn, Richard Dano, Carolin Höcklmann, Katrin Balzer, Sascha Köpke, Martin Scherer, Eva Hummers, Christiane Müller

**Affiliations:** University Medical Center Goettingen, Göttingen, Germany

**Keywords:** Interprofessional relations, home healthcare, family, health professions

## Abstract

**Background:**

About one million people are in need of healthcare at home in Germany. This is a mostly complex endeavour because interprofessional collaboration is often challenging. This might have a negative impact on patient safety.

**Research questions:**

How do people receiving home care (PRHC), relatives, registered nurses from home care services, general practitioners (GP) and therapists (occupational-, physio-, speech therapists) perceive interprofessional collaboration in the home care setting?

**Method:**

Semi-structured interviews were conducted with 20 PRHC and 21 relatives. Additionally, we performed nine monoprofessional focus groups involving nurses of home care services (*n* = 17), GP (*n* = 14), and therapists (*n* = 21). Data were analysed by qualitative content analysis.

**Results:**

Three main categories were identified: (a) ‘perception of interprofessional collaboration’, (b) ‘means of communication’, and (c) ‘barriers and facilitators’. PRHC and relatives often perceive little to no interprofessional collaboration and take over a significant part of the organisational coordination and information exchange. Interprofessional collaboration in steady care situations does exist at times and mostly concerns coordination tasks. Contact and information exchange are rare. However, interprofessional personal encounters are sporadic. Fixed agreements and permanent contact persons are not standard but increase with the complexity of the healthcare situation. Joint collaborations are often perceived as highly beneficial. Means of communications such as telephone, fax, or E-mail are used differently and are often considered tedious and time-consuming. In general, no interprofessional formal written or electronic documentation system exists. Personal acquaintance and mutual trust are beneficial, while a lack of mutual availability, limited time, and inadequate compensation hinders interprofessional collaboration.

**Conclusions:**

Interprofessional collaboration in-home care occurs irregularly, and coordination often remains with PRHC or relatives. While this individual care setup may work relatively straightforwardly in care situations of low complexity, it becomes vulnerable to disruptions with increasing complexity. Close interactions, collaboration, and fixed means of communication might improve healthcare at home.

# Abstract: How do Norwegian GPs experience participation in mandatory groups for continuing medical education?

**Authors:** Torunn Bjerve Eide

**Affiliations:** Dept of General Practice, University of Oslo, Oslo, Norway

**Keywords:** Continuing medical education, quality improvement, professional update, mixed-methods

## Abstract

**Background:**

To ensure high quality services in general practice, we need both a comprehensive specialist education as well as evidence-based professional development after specialisation. Norwegian GP specialists participate in mandatory Continuing Medical Education (CME) groups. We lack research on the GPs experience and evaluation of these groups.

**Research questions:**

How do current CME groups function both organisationally and in terms of content? What do GPs perceive as the groups’ most important functions and needs for improvement?

**Method:**

All Norwegian GPs were invited to participate in the study. A questionnaire was developed and piloted by experienced general practitioners and researchers. The questionnaire includes both closed-ended and free-text questions. Collected data will be analysed quantitatively and with thematic analysis as described by Braun and Clarke.

**Results:**

Five hundred and seventy-nine GPs answered the questionnaire, 59% were female, and 89% were GP specialists. The CME groups consisted of from 2 to 17 participants (median 6). Thirty-eight per cent of the groups consisted of GPs working together, while the remaining groups consisted of GPs from different practices. The mean time of participation in the same CME groups was 10 years (range 0 to 45). Almost 91.5% of respondents were happy or very happy with the total experience in their CME group. The groups were seen as important not only as a compulsory activity but also for quality improvement, professional updates and even a social setting. Many expressed that the groups gave essential support when dealing with professionally difficult issues. Some called for an idea bank for subjects to discuss in the groups, however, many emphasised that the groups functioned very well as they were.

**Conclusions:**

Norwegian GPs reported very positive experiences with their mandatory CME groups. As the GPs everyday work can be relatively lonely, the groups role as a professional and emotional sounding board was seen as important. Further findings will be presented at the congress.

# Abstract: Characterisation, symptom pattern and symptom clusters from a retrospective cohort of long COVID patients in primary care in Catalonia

**Authors:** Gemma Torrell, Diana Puente, Constanza Jacques Aviñó, Israel Rodriguez-Giralt, Lucía Amalia CarrascoRibelles, Concepció Violán Flors, Veronica Royano, Alba Molina Cantón, Tomás López Jiménez, Laura Medina Perucha, Anna Berenguera

**Affiliations:** Institut Català de la Salut, Barcelona, Spain

**Keywords:** SARS-CoV-2, long COVID, post-COVID-19 syndrome, cluster analysis, primary health care, participatory research

## Abstract

**Background:**

Around 10% of people infected by SARS-COV-2 report symptoms that persist longer than 3 months. Little has been reported about sex differences in symptoms and clustering over time of non-hospitalised patients in primary care settings.

**Research questions:**

What are the characteristics and evolution of symptoms over time in patients with Long COVID visits at primary care settings in Catalonia?

**Method:**

Descriptive study of a primary care cohort of patients with symptoms’ persistence ≥3 months from clinical onset in co-creation with the Long Covid Catalan group using an online survey. Recruitment: March 2020–June 2021. Exclusion criteria: being admitted to ICU, <18 years old, not living in Catalonia. 117 symptoms were gathered in 18 groups. A cluster analysis was performed at 21 days of infection (baseline), 22–60 days and ≥3 months.

**Results:**

We analysed responses of 905 participants (80.3% women). General symptoms were the most prevalent with no differences by sex, age, or wave. Its frequency decreased over time. Dermatological (52.1% in women, 28.5% in men), olfactory (34.9%, 20.9%) and neurocognitive symptoms (70.1%, 55.8%) showed the greatest differences by sex. Cluster analysis showed five clusters with a predominance of ‘Taste & smell’ (24.9%) and ‘Multisystemic’ clusters (26.5%) at baseline and’ Multisystemic’ (34.59%) and ‘Heterogeneous’ (24.0%) at ≥3 months. The ‘Multisystemic’ cluster was more prevalent in men. The ‘Menstrual’ cluster was the most stable. Most transitions occurred from ‘Heterogeneous’ cluster to ‘Multisystemic cluster’ and from ‘Taste & Smell’ to ‘Heterogeneous’.

**Conclusions:**

General symptoms were the most prevalent in both sexes at three-time cut-off points. Major sex differences were observed in dermatological, olfactory and neurocognitive symptoms. The increase of the ‘Heterogeneous’ cluster might suggest an adaptation to symptoms or a non-specific evolution of the condition, which can hinder its detection at medical appointments. A careful symptom collection and patients’ participation in research may generate useful knowledge about Long Covid presentation in primary care settings.

# Abstract: Exploration of sexuality in women after sexual violence

**Authors:** Emeline Pasdeloup, Chloé Duboc, Mathilde Leblanc

**Affiliations:** Faculté de Médecine de Tours, Ouzouer sur Loire, France

**Keywords:** Sexual violence, sexuality, women

## Abstract

**Background:**

In France, 15% of women report having been the victim of at least one sexual assault in their lifetime. The impact of such violence on health in general is well known, but the consequences for sexual health are poorly assessed. We can therefore ask whether such violence has an impact on women’s sexuality, and whether taking their sexual health into account would improve their quality of life.

**Research questions:**

Exploring the sexuality of women victims of sexual violence after the violence.

**Method:**

Recruitment for this qualitative study followed a maximum variation sample strategy. Six MINI (McGill Illness Narrative Interview)-inspired semi-structured interviews were conducted. They were transcribed and analysed using a method inspired by grounded theorising.

**Results:**

The participants were all vulnerable before the violent incident. After the incident, they describe negative cognitions, a negative body image and relationship difficulties, all of which contribute to the onset of sexual difficulties. To regain a fulfilled sexuality, they all express the need for healing, aided by the help of loved ones or professionals. But it can also be hampered by various obstacles the main one being the lack of proactive exploration of sexuality matters by healthcare professionals.

**Conclusions:**

These results are in line with the literature on the main sexual difficulties encountered by victims of sexual violence. They highlight the patients’ expectation that healthcare professionals should proactively explore this subject. It would be interesting to know what inhibits professionals from approaching this subject, and whether there are any specific aspects to be taken into account with female victims of sexual violence. This suggests the need to improve awareness among the professionals who meet these women, and highlights the need to provide them with more practical tools and training.

# Abstract: Cardiovascular risk and cognitive performance: A population-based cross-sectional study (NEDICES2-RISK)

**Authors:** Javier Rubio Serrano, Beatriz Arregui-Gallego, Ileana Gefaell, Ester Tapias-Merino, Maria Del Canto De Hoyos-Alonso, Isabel Del Cura

**Affiliations:** FIIBAP, Madrid, Spain

**Keywords:** Heart disease risk factors, neuropsychological tests, cognitive dysfunction, clinical decision rules

## Abstract

**Background:**

Cardiovascular risk (CVR) factors can increase the likelihood of cognitive impairment. CVR functions seem to be a reliable predictor of this risk.

**Research questions:**

Is there a relationship between CVR and cognitive performance in the Spanish population?

**Method:**

Cross-sectional study of a community-based cohort, aged 55 to 75 years with no previous cardiovascular events. The study estimated CVR using the Framingham REGICOR (Registre Gironí del Cor) and FRESCO (Función de Riesgo Española de acontecimientos Coronarios y Otros) equations. Participants underwent neuropsychological tests to explore the following cognitive domains: global cognition, memory, premorbid intelligence, verbal fluency, visuoconstruction, attention, and psychomotor speed. A generalised linear model was fitted to analyse the influence of CVR on cognitive performance for each risk function.

**Results:**

The study included 859 participants The median age was 67 (IQR: 62.00–71.0) years, and 56.2% were women. Women with higher CVR, as determined by REGICOR, had lower scores in the Trail Making Test A part 2 (TMTA-2) with an odds ratio (OR) of 4.35, (CI95% 1.16-18.45). High CVR by FRESCO, showed lower scores in the Immediate OR:5.16 (CI95% 1.91-15.64), and Delayed Recall tests OR:2.75, (CI95% 1.05-7.61), and TMTA part 1 (TMTA-1) OR:6.77 (CI95% 2.13-21.61). Men with higher CVR estimated with the REGICOR function had lower scores in the TMTA-1 OR:3.14 (CI95% 1.30-7.66), and TMTA-2 OR:2.75 (CI95% 1.18-6.44). Similarly, those with moderate CVR exhibited poorer scores in TMTA-1 OR:2.16 (CI95% 1.09-4.45). The FRESCO function also indicated that men with moderate CVR presented lower scores in the Immediate OR:2.23 (CI95% 1.20-4.19) and Delayed Recall tests OR:2.35 (CI95% 1.24-4.49), as well as TMTA-1 OR:2.49 (CI95% 1.14-5.55).

**Conclusions:**

Despite the absence of previous cardiovascular disease, poor cognitive performance was associated with high CVR in men and women. Memory, attention and psychomotor speed were the most affected cognitive domains.

# Abstract: How children and parents experience home-based guided hypnotherapy: A qualitative study

**Authors:** Ilse Ganzevoort, Adriëlla Van Der Veen, Manna Alma, Marjolein Berger, Gea Holtman

**Affiliations:** University Medical Center Groningen, Groningen, The Netherlands

**Keywords:** Qualitative study, children, functional abdominal pain, hypnotherapy, primary care

## Abstract

**Background:**

A general practitioner (GP) sees many children with functional abdominal pain, gives education and reassurance, but has no treatment to offer. Home-based guided hypnotherapy could be a treatment option, but the experiences of patients have not been studied. Insight in experiences could guide implementation in primary care and optimise its use.

**Research questions:**

How do children and parents experience home-based guided hypnotherapy for functional abdominal pain?

**Method:**

This study is part of a randomised controlled trial evaluating the (cost-)effectiveness of home-based guided hypnotherapy in primary care. For this qualitative study, we used open-ended questions from the trial questionnaire. We used purposive sampling to invite children and parents for a semi-structured in-person interview. Parents of children with low adherence were interviewed by telephone. Interviews were audio recorded, transcribed verbatim and coded by two authors. We used thematic content analysis to analyse the data.

**Results:**

Questionnaire data from 54 participants were collected we interviewed 10 child-parent dyads (20 interviews total), and three parents of children with low adherence. Children were 7–13 years old. Three themes emerged: child, environment, and website. Children’s experience was influenced by their age, character, imagination and creativity, and whether they felt any effect. Children enjoyed hypnotherapy when they experienced less abdominal pain or better sleep but disliked it when they were unable to surrender to the exercises. The environment (e.g. parent involvement, time) and the website (e.g. looks, voice, functionality) influenced participants’ experiences and adherence. Participants would like personalised therapy regarding exercise duration and level, looks, voice and a reward system.

**Conclusions:**

This study showed that many children feel positive effects, but their experiences of hypnotherapy vary. Insights provided by this study aid in developing a hypnotherapy application suited to the child’s preferences and needs. Such an accessible treatment option could help GPs in managing children with functional abdominal pain in daily practice.

# Abstract: Continuity of care in general practice: A survey among the general practitioners in Finland

**Authors:** Nina Tusa, Ulla Mikkonen, Kadri Suija, Tuomas Koskela, Pekka Mäntyselkä

**Affiliations:** University of Eastern Finland, Kuopio, Finland

**Keywords:** Continuity of care, primary health care, general practice

## Abstract

**Background:**

Continuity of care (COC), defined as a longitudinal relationship with a personal physician, is one of the core values of general practice. In the Finnish healthcare system, however, maintaining it might be challenging.

**Research questions:**

The aim of this study was to analyse how continuity of care is realised in the opinion of general practitioners (GPs) and what practices could be used to implement and improve it.

**Method:**

We conducted a web-based survey among GPs in Finland from May to October in 2023. The questionnaire was developed by the study team and added to the Webropol online environment of the University of Eastern Finland. An invitation and link to the online questionnaire were sent by e-mail to the contact people of the wellbeing services, requesting it to be forwarded to the GPs working in their area. We analysed data using inductive thematic analysis.

**Results:**

Altogether, 432 questionnaires were answered. The participants were evenly spread across Finland, 54% of them were specialists in general practice and 69% had more than 5 years of working experience as a GP. Regarding the questions of existing practices and how to improve COC, GPs mentioned the following codes: a flexible appointment system and arrangement of follow-up care; improving the assessment of the need for treatment; targeting COC for special patient groups; smaller centres, which support familiarisation; the practical and flexible ways of organising work; good leadership with a shared aim to routinely COC. Having enough healthcare workers and stable staff were seen as prerequisites for improving COC. GPs associated COC with the meaningfulness of work and well-being. The results of the theme analysis will be presented in the congress.

**Conclusions:**

The GPs identified existing practices that support COC as well as suggested approaches to improve it in general practice.

# Abstract: Pneumococcal vaccination in risk groups – the reality of a family health unit in Portugal

**Authors:** Carolina Pais Neto, Bárbara Duarte Ferreira, Carla Almeida

**Affiliations:** USF Novo Norte – ULS Entre Douro e Vouga, Arouca, Portugal

**Keywords:** Pneumococcal vaccine, pneumococcal infection, prevention, vaccination, primary health care

## Abstract

**Background:**

Pneumococcal disease is an important cause of morbidity and mortality potentially preventable through vaccination. Despite the recommendation for vaccination in adults belonging to increased risk groups for invasive pneumococcal disease (IPD), it remains underused worldwide.

**Research questions:**

We aimed to assess the pneumococcal vaccination profile in risk groups within a Portuguese Family Health Unit.

**Method:**

We conducted an observational cross-sectional and descriptive study among adults registered in a Portuguese Family Health Unit in July 2023, aged ≥65 years or ICPC-2 coding corresponding to risk conditions for IPD, by the Portuguese clinical guidance standard, based on WHO recommendations.

**Results:**

From the 2463 subjects identified as at-risk adults, a representative sample of 333 patients was obtained, 53.8% female and with a mean age of 72 years (minimum = 19; maximum = 97). The pneumococcal vaccination rate was 24.6%, of which 58.5% had incomplete vaccination. The predominant vaccination scheme was the 13-valent conjugate vaccine followed by the 23-valent polysaccharide vaccine (90.3%), with 65% prescribed in primary health care. Vaccination rate varied according to the IPD risk group, being higher in adults with a greater number of risk factors (*p* = 0.023). There was also a trend towards increased vaccination in the absence of economic insufficiency (*p* = 0.327) and at higher levels of education (*p* = 0.413).

**Conclusions:**

The reduced pneumococcal vaccination coverage in risk groups and the prevalence of incomplete vaccination requires a reflection on prescription and vaccination adherence determinants related to the doctor and the patient, as a means to implement effective measures aimed at improving the quality of pneumococcal vaccination in this population.
